# Supplementary material for: The mediating role of self-efficacy in the relationship between self-management and health-promoting behaviors in post-PCI patients
Source: PLoS One. 2026 Jan 29;21(1):e0341826. doi: 10.1371/journal.pone.0341826 (PMC12854456; doi:10.1371/journal.pone.0341826)
Supplement: Attachment — Submitted filename: Reviewer Comment- PLOS 6TH OCT.docx [file pone.0341826.s001.docx]

- **Reviewer Comment**: **The mediating role of self-efficacy in the relationship between self-management and health-promoting behaviors in post-PCI patients**


  **Typos/Grammatical Errors:**
- **Page 1, Line 17**: Remove “a”: “health promoting behaviors
- **Page 4, Line 111**-113: Sentences are fragmented due to line breaks (e.g., “behaviours.The Cronbach’s alpha...”)
- **Page 11, Line 224**: “wh ich is favourable”
- **Page 14, Line 258:** “patien ts'”
- **Page 16, Line 292:** “patients. Furthermore, targeted interventions may be implemented…”- Combine for better flow of the sentence

**Narrative:**

- **Page 2-3, Line 36-84**: The introduction effectively presents the rationale but could be tightened for clarity. Several ideas overlap (e.g., repetitive mention of CAD burden and PCI limitations).
- **Page 11-13, Line 199-257:** The discussion section largely restates results rather than interpreting them deeply. It would benefit from connecting findings to specific mechanisms (e.g., how self-efficacy transforms motivation into lifestyle adherence).
- **Page 15, Line 281-289:** Well-structured limitation portion but needs more analytical reflection.
- **Page 13-15**: Effect sizes (γ-values) and mediation proportion should be discussed more critically. Eg disucss why 23.5% mediation is clinically meaningful

**Methods/Results**:

- **Page 4-6**: Sampling and scale sections are clear but lack justification for validity
- **Page 9-10:** Data representations seems to be repetitive between text and tables
